# Periventricular Heterotopia: Shuttling of Proteins through Vesicles and Actin in Cortical Development and Disease

**DOI:** 10.6064/2012/480129

**Published:** 2012-10-22

**Authors:** Volney L. Sheen

**Affiliations:** Department of Neurology, Beth Israel Deaconess Medical Center and Harvard Medical School, Boston, MA 02115, USA

## Abstract

During cortical development, proliferating neural progenitors exhibit polarized apical and basolateral membranes that are maintained by tightly controlled and membrane-specific vesicular trafficking pathways. Disruption of polarity through impaired delivery of proteins can alter cell fate decisions and consequent expansion of the progenitor pool, as well as impact the integrity of the neuroependymal lining. Loss of neuroependymal integrity disrupts radial glial scaffolding and alters initial neuronal migration from the ventricular zone. Vesicle trafficking is also required for maintenance of lipid and protein cycling within the leading and trailing edge of migratory neurons, as well as dendrites and synapses of mature neurons. Defects in this transport machinery disrupt neuronal identity, migration, and connectivity and give rise to a malformation of cortical development termed as periventricular heterotopia (PH). PH is characterized by a reduction in brain size, ectopic clusters of neurons localized along the lateral ventricle, and epilepsy and dyslexia. These anatomical anomalies correlate with developmental impairments in neural progenitor proliferation and specification, migration from loss of neuroependymal integrity and neuronal motility, and aberrant neuronal process extension. Genes causal for PH regulate vesicle-mediated endocytosis along an actin cytoskeletal network. This paper explores the role of these dynamic processes in cortical development and disease.

## 1. Cortical Development

The development of the mature six-layered cerebral cortex involves a highly ordered and complex sequence of events that requires tight control of neural progenitor cell proliferation, cell fate specification, and initial migration from their site of origin. Neuroblasts must then migrate to their final destination and undergo region-specific differentiation. Many excitatory neurons within the cerebral cortex originate from the pseudostratified ventricular epithelium that lines the embryonic cerebral ventricle wall [1]. The ventricular epithelium is composed of bipolar neural progenitor cells that span the ventricular to pial surface, so-termed neuroepithelial cells. Neuroepithelial cells exhibit characteristic epithelial features including the polarized localization of transmembrane proteins along their apical plasma membrane (prominin-1) and the localization of adherens junctions along their basolateral plasma membrane [2]. At first, these cells undergo several rounds of symmetric cell division to produce two identical daughter cells that continue to proliferate [1]. The consecutive rounds of symmetric cell division result in the generation of increasing numbers of neural progenitors and rapid expansion of the ventricular zone thereby enlarging the surface area of the cortex [3]. At the onset of neurogenesis, which occurs approximately during the fifth week of gestation in humans (or embryonic day 11 in mice) some neuroepithelial cells adopt an asymmetric mode of cell division [4, 5]. In this asymmetric mode of cell division, one daughter cell retains characteristics of the parent cell and continues to proliferate while the other daughter cell does not undergo mitosis but rather migrates towards the pial surface of the cortex [1]. The transition from symmetric to asymmetric cell division (shift in cell fate and decision to initiate migration) coincides with an increase in cortical thickness [3]. Neural progenitors which initially migrate from the VZ and adopt positions in the subventricular or intermediate zones may undergo several additional cycles of amplification, generating either pairs of new neurons or pairs of intermediate progenitors [6–8]. As neurogenesis continues, neuroepithelial cells downregulate their epithelial characteristics to form a new type of progenitor cell called radial glial cells. Radial glial cells display residual neuroepithelial as well as astroglial properties. Similar to neuroepithelial cells, radial glial cells extend long bipolar processes from the ventricle to the pial surface. In contrast to neuroepithelial cells, radial glial cells express several molecules commonly expressed by astrocytes including astrocyte-specific glutamate transporter (GLAST) and glial fibrillary acidic protein (GFAP) [2, 9]. Most neurons within the brain are generated either directly or indirectly from radial glial cells, although a subpopulation derives from intermediate progenitors during the middle and later stages of cortical development [10].

Neuronal migration in humans occurs between gestational age of 10–20 weeks and immediately follows neurogenesis [4]. During this process, neurons born within the germinal zones (ventricular zone and ganglionic eminences) migrate radially towards the pial surface resulting in the formation of six layers of neurons within the cortical plate [1]. Early birth-dating studies using tritiated thymidine have shown that neurons accumulate within their respective layers in an “inside-out” sequence according to their relative birth-date [11, 12]. These studies revealed that cells born at subsequent later stages of cortical development migrate past earlier born cells rather than merely displacing them outward towards the pial surface. As a result of this “inside-out” pattern of neuronal migration, the earliest-born neurons are located within the deepest cortical layers while the latest born neurons are found superficially. The formation of cortical layers begins after the first neurons generated within the ventricular zone migrate towards the pial surface. Neurons born from the earliest rounds of neurogenesis will migrate out of the ventricular zone to form a layer of cells beneath the pial surface called the preplate. Neurons born from the next round of neurogenesis will then enter the preplate splitting this layer into a superficial marginal zone and an underlying subplate. Finally, neurons born from subsequent rounds of neurogenesis will migrate past the subplate to stop just below the marginal zone which will form layer I of the cortical plate [13]. The various neocortical laminae (layers II–VI) are formed as successive waves of migrating neurons reach the cortex. A series of electron microscopic and tracing studies have revealed that cells may employ radial or tangential migratory trajectories in route to the cortical plate. Initial serial section electron microscopic studies showed that the entire length of migrating neurons remains closely juxtaposed to radial glial fibers that span the width of the cortex. Based on this finding, early born neurons were thought to migrate radially along radial glial scaffolding before reaching their designated positions within the developing cortical plate [14]. This radial mode of neuronal migration was subsequently verified in real-time-imaging studies of dye-labelled migrating cells within the cortex [15]. Subsequent studies indicate that neurons also migrate tangentially to the cortex. Retroviral tracing studies suggested that clonally related cells disperse widely rather than remain along a single radial glial scaffold [16]. Imaging studies of dye-labelled cells within the cortex revealed that both postmitotic and precursor cells disperse tangentially across radial glial fibers during neuronal migration [15, 17, 18]. More recent studies have shown that virtually all interneurons of the cerebral cortex are produced in the ganglionic eminence of the basal forebrain and arrive in the cortex in a ventral-to-dorsal tangential migratory pathway [19, 20]. Once interneurons reach the cortex, they adopt both radial and oblique routes towards their final destination within the cortical plate [21]. Ultimately, this wave of migratory neurons forms the various cortical layers: (II) the external granular layer with small pyramidal neurons and numerous stellate neurons, (III) the external pyramidal layer, containing small- and medium-size pyramidal neurons, as well as nonpyramidal neurons, (IV) the internal granular layer with stellate and pyramidal neurons that receive thalamocortical afferents, (V) the internal pyramidal layer comprised of large projection pyramidal neurons that send signals to the basal ganglia and spinal cord, and (VI) the multiform layer consisting of pyramidal, spindle-like, and multiform neurons which send efferents to the thalamus. 

## 2. Malformations of Cortical Development

Disruptions in one or more aspects of corticogenesis (proliferation, migration, and differentiation) can give rise to a group of abnormal brain developmental syndromes called cortical malformation disorders. These disorders have recently been reclassified into subgroups based upon their anatomical abnormalities and the shared pathways and mechanisms of action for the specific classifications: malformations secondary to abnormal neuronal and glial proliferation or apoptosis, malformations due to abnormal neuronal migration, and malformations secondary to abnormal postmigrational development [22]. 

In a simplistic view, initial cortical development requires expansion and generation of sufficient neural progenitors. Many genes causal for proliferative disorders such as the primary microcephalies (small brain) are expectedly involved in various pathways implicated in cell cycle and cell division including transcriptional regulation, cell cycle progression and checkpoint regulation, centrosome duplication and replication, mitotic spindle formation, and DNA repair [23]. More focal dysplasias (abnormal focal growth of cells) appear to involve genes that regulate the integration of various cues such as growth factors, cell cycle signals, and nutrients that can direct proliferation of smaller cell populations [24]. 

Following expansion of the progenitor pool, cells must migrate into the cortical plate. Abnormalities in neuronal migration have been thought to give rise to periventricular heterotopia (nodules of neurons aligned along the lateral ventricles), subcortical heterotopias (ectopic clusters of neurons typically localized between the ependyma and the cortex), and lissencephalies (disruption in migration along the entire extent of the cortical width). Taken in the context of the anatomical abnormality, periventricular heterotopia has been viewed as a disruption in initial neuronal migration due to disruption of the ventricular neuroepithelium [25]. Loss of ependymal integrity may cause disengagement of radial glial end feet along the denuded epithelium, thereby preventing early postmitotic neurons from migrating into the cortical plate. Genes causal for this disorder play some role in actin regulation and vesicle trafficking [26–29]. Widespread disruption of transmantle migration can lead to gross disruption of lamination and cortical folding with agyria, widened sulci, and gyri formation as in pachygyria and subcortical band heterotopia. Not surprisingly, many genes that lead to disruption of neuronal migration and motility appear to play some role in microtubule function [30–32]. 

Following migration, neurons which reach the cortical plate but develop abnormally can give rise to some poorly understood cortical malformations. Polymicrogyria (referring to multiple small gyri along the cortical surface) and schizencephaly (characterized by grey matter tissue extending from the ependymal lining of the cerebral ventricles to the pial surface of the cerebral hemisphere surface) are heterogeneous disorders. The underlying pathophysiology is poorly understood.

## 3. Periventricular Heterotopia

Periventricular heterotopia (PH) refers to a malformation of cortical development characterized by bilateral near-contiguous ectopic neuronal nodules found along the lateral ventricles (so-termed periventricular heterotopia) [33]. The nodules are thought to reflect impaired migration from the VZ. Some individuals harboring mutations in the causative genes also have microcephaly (meaning small brain), suggestive of impaired neuronal generation or increased cell death [26, 34]. Overall, these observations suggest that genes involved in PH formation play some role in migration (heterotopia formation) and proliferation (microcephaly).

The anatomical localization of this disorder raises interesting implications for genes that are causal for this disorder. During normal cortical development, neural progenitors reside along the VZ, undergo progressive expansion through proliferation, and then undergo cell fate specification either to remain as a progenitor, transform into an intermediate progenitor or assume a postmitotic neuronal fate. Intermediate progenitors and postmitotic neurons must undergo initial migration from the VZ toward the cortical plate, whereas progenitors reenter the cell cycle. Interactions between progenitors and the extracellular environment and regulation of cell cycle proteins are going to influence the developmental fate of neural progenitors. Given that PH reflects a developmental defect specifically within this population of cells during a fairly confined temporal framework, genes involved in this disorder will in all likelihood play a significant role in regulating neural progenitor proliferation and migration.

## 4. Genetics Underlying PH Formation

Neurogenetic analyses have identified two human genes are known to cause PH formation. The most common form of PH is inherited in an X-linked dominant fashion from mutations in the *FLNA *gene [28]. Filamin A encodes the cytoplasmic actin-binding protein FLNA, which serves as a scaffold to over thirty proteins [35]. Given FLNA interactions with the actin cytoskeleton, PH has been thought to arise from a disorder in neuronal motility. Through X-inactivation, cells harboring the mutant FLNA protein would fail to migrate from the ventricular zone, whereas cells expressing the normal FLNA protein undergo normal migration into the cortex [28]. A rare form of autosomal recessive PH with microcephaly (ARPHM) has been associated with mutations in the *ARFGEF2* gene. *ARFGEF2* encodes Brefeldin-A inhibited Guanine Exchange Factor-2 (BIG2) [26]. BIG2 is a protein kinase A anchoring protein (AKAP) which regulates Golgi-vesicle trafficking through its Sec7 domain. Although FLNA and BIG2 proteins appear to carry out different functions, similar radiographic findings of PH suggest that they might be involved in the same molecular pathway important in neural progenitor development. Moreover, since PH due to *ARFGEF2 *mutations is an autosomal recessive disorder, all migratory neurons harbor the gene mutation. Thus, it is unlikely that a cell motility problem alone causes this malformation because only some and not all the neurons fail to migrate from the VZ into the cortical plate. 

Other genetic abnormalities have been associated with this disorder. Anterior PH has been reported with duplication of human chromosome 5p15 [36]. Diffuse but variable PH is caused by a deletion on 6q27 [37], whereas PH with diffuse white matter changes is associated with 6p25 deletion [38]. PH and William's syndrome have been identified and involved a deletion on 7q11.23 and include the region spanning the *HIP1* and *YWHAG* genes [37, 39]. Deletion of chromosome 1p36 gives rise to PH and agenesis of the corpus callosum [40]. Genomic deletions leading to PH have also been reported to localize to 4p15, 5q14 [41, 42]. Finally, triplet CGG nucleotide repeat expansion of the *FMR1* gene can lead to PH in Fragile X [43], as can mutations in the polyglutamine repeat binding protein *PQBP*1 [44]. Triplet repeat disorders have similarly been implicated in vesicle trafficking, suggesting a shared common pathway in the human PH phenotype [45].

Several mouse genes have been associated with PH formation. Stem-cell-factor- (SCF-) c-kit regulates proliferation and migration of neural progenitor cells. Direct administration into the ventricles during cortical development led to neuronal and glial heterotopia as well as disruption of the neighboring cytoarchitecture [46].

Deletion of the rho GTPases Cdc42 also disrupts local adherens junctions and proliferation of basal progenitors leading to heterotopia formation through both impaired intranuclear migration and disruption of the neuroependymal lining [47]. Spred1, a negative regulator of Ras-MAPK-ERK, similarly leads to PH formation. In cortical progenitor cells, Spred1 localizes within distinct vesicles, indicating a potential role in transport [48]. Mekk4 binds FlnA and regulates the CSBP2 and JNK MAPK (but not the mitogen-activated ERK MAPK) pathways which are activated by environmental stresses such as osmotic shock, UV irradiation, wound stress, and inflammatory factors [49]. PH is also seen in mutations in the *Napa* gene, which encodes for soluble N-ethylmaleimide-sensitive factor (NSF) attachment protein alpha (alpha Snap) and is involved in SNAP receptor- (SNARE-) mediated vesicle fusion [25, 50].

## 5. Filamin A and PH

### 5.1. Genetic Phenotype

While mutations in *FLNA* are the most common cause of inherited PH in the central nervous system (CNS) [51], it has become increasingly clear that disruptions in the function of this actin-binding protein are not limited to the brain. Understanding its function both inside and outside the CNS will provide insight into fundamental role that this and associated interacting proteins play in PH, its related phenotypes, and ultimately neural progenitor development.

In the CNS, familial PH is associated with an X-linked dominant inheritance pattern such that males are hemizygous lethal and females present with the classical PH phenotype. In females, X-inactivation leads to a proportion of cells which normally expresses FLNA whereas others have no protein expression. In addition to the bilateral heterotopic nodules, the disorder is associated with thinning of the corpus callosum and an enlarged cistern magna. Females can develop varying degrees of epilepsy and dyslexia, as well as worse adaptive skills and conduct problems [52, 53]. The complete loss of FLNA phenotype in males is more severe with a thinned but normal six layered cortex. Polymicrogyria, thinning of the corpus callosum, enlarged ventricles, and reduced white matter are seen in hemizygous males. The brainstem and cerebellum are unaffected [54, 55].

Mutations in FLNA have been increasingly recognized to affect extra-CNS organ systems in giving rise to phenotypic heterogeneity [56, 57]. Robertson identified mutations in the actin-binding gene as causal for four skeletal dysplasias (otopalatodigital syndrome OPD types 1 and 2, frontometaphyseal dysplasia, and Melnick-Needles syndrome) with abnormalities in craniofacial structures, skeleton, brain, viscera, and urogenital tract. Mutations in FLNA clustered into four regions of the gene, the actin-binding domain and rod domain repeats 3, 10, and 14/15, suggesting some type of gain of function phenotype [58, 59]. However, an in-frame deletion on repeat 24 also produces PH with similar skeletal changes suggestive of OPD associated with flat face and spatulate finger tips, short broad phalanx and metacarpus, and bowed radius with dislocated wrist joints in males, suggesting that this may not just be a gain of function mechanism [60]. FLNA mutations have also been associated with cutaneous manifestations such as terminal osseous dysplasia (exon 31) and the connective tissue disorder, Ehlers-Danlos syndrome (EDS) [27, 61, 62]. Cardiac valvular disease (repeat 1, 4, and 5) and vascular abnormalities are commonly seen in this disorder. FLNA mutations typically give rise to aortic or mitral regurgitation, coarctation of the aorta, or other left-sided cardiac malformations [63, 64]. Moreover, the early embryonic lethality seen in null FLNA mutations in males likely arises from excessive bleeding due to a vasculopathy. FLNA plays a role in lung development and loss of function has been reported to cause lobar emphysema, bilateral atelectasis, lung cysts, and tracheobronchomalacia, leading to pulmonary arterial hypertension and long-term oxygen dependence [65, 66]. The defects appear in-part secondary to defects in the formation of bronchial cartilage. Finally, FLNA dysfunction and PH have been linked to intestinal pseudoobstruction. Pathology showed abnormal lamination of the small intestinal muscularis propria and multinucleated myocytes [67–69]. Individuals with the pseudoobstruction also variably presented with patent ductus arteriosus, and thrombocytopenia with giant platelets, further reiterating the broad multiorgan system involvement of FLNA [69].

Several observations can be drawn from the various phenotypes reportedly due to FLNA mutations. First, heterotopiae are not the only anatomical malformation seen in the CNS with this disorder. The reduced cortical layer width and mental retardation seen in males with this disorder implicates FLNA in neural progenitor proliferation. The thinning of corpus callosum, dyslexia, and epilepsy also argues that FLNA is likely involved in neuronal process extension and formation. Second, FLNA is broadly expressed throughout the body and its phenotypes do not always appear to be fully penetrant, suggesting that there are extrinsic factors which modify the clinical presentation. Third, some shared features are seen in the abnormalities within the different organ systems. As with the brain where there appears to be disruption of the cell connectivity and adhesion along the neuroependyma [25], weakening of skin integrity is seen in EDS, the vasculopathy has been reportedly due to leakiness in vessels from loss of vascular endothelium, and the emphysema and cyst formation in the lungs also could implicate loss of cell adhesion.

### 5.2. Structure

FLNA represents one of three members (FLNA, FLNB, FLNC) of the filamin family of actin-binding proteins. The filamin proteins share a high degree of homology between the conserved exon/intron structure with significant differences in exon 32 of all paralogs encoding the hinge I region, as well as the insertion of a novel exon 40A in FLNC only [70]. Moreover, these proteins are shown to physically interact and heterodimerize, potentially suggesting a mechanism with which to regulate FLNA function [71].

FLNA is a 280 kD actin-binding phosphoprotein represented by an N-terminal actin-binding domain, followed by Immunoglobulin- (IG-) like repeat domains, that contains the receptor binding region at the C-terminus. FLNA associates with itself to form a homodimer that can regulate the actin cytoskeleton through interactions derived from its multiple receptor binding regions, thereby directing cell stability, protrusion, and motility [72, 73]. Filamin also promotes actin branching, tethers large actin filaments, and holds them in a perpendicular arrangement [74, 75]. The resulting three-dimensional orthogonal network of actin filaments represents a characteristic cortical actin structure at the leading edge of migrating cells. Lastly, FLNA contains a PKA-dependent phosphorylation site at serine 2152 that has been shown to regulate its redistribution to the cell membrane [76].

### 5.3. Function

FLNA has been shown to interact with numerous other proteins, suggesting many potential mechanistic roles that could influence cortical development. These interactors demonstrate great diversity but can be clustered into several general functional groups including (1) interactions with transmembrane receptors and signaling molecules such as *β*-integrin, dopamine, and G-protein-coupled calcium sensing receptors, (2) signaling through diverse intracellular cell signaling kinases, phosphatases, and adaptor molecules such as SHIP-2 or SEK1, and (3) regulation of cortical actin networks through molecules including the Rho family of small GTPases [35, 77–80].

FLNA has been reported to interact with a vast array of cell surface receptors. For example, FlnA interactions with integrins provide a link between the extracellular matrix and regulation of the actin cytoskeleton [81]. FlnA also binds dopamine and epidermal growth factor receptors, suggesting some role in signal transduction of secreted neurotransmitters and growth factors [82, 83]. The androgen receptor also interacts with the actin-binding protein and the two proteins colocalize in the nucleus implicating FLNA in transcriptional regulation [84, 85]. In the absence of FLNA binding, the cystic fibrosis transmembrane conductance regulator is rapidly internalized from the cell surface, where it accumulates prematurely in lysosomes and is ultimately degraded [86]. Similar FLNA-dependent lysosomal degradation is seen with the FcgammaRI, a high-affinity IgG, and recycling of calcitonin receptors [87, 88]. These observations indicate that FLNA not only serves as a means for signal transduction from receptors onto the actin cytoskeleton and associated signaling components but also suggests that the protein actively regulates the turnover and stabilization of receptors.

Following receptor activation, FLNA may alter the cortical actin cytoskeleton. In general, FLNA is thought to bind and activate RhoGTPases, thereby regulating their consequent effectors involved in actin dynamics. CD4 and its coreceptors interact with FLNA to direct both the clustering of these HIV receptors on the cell surface but also mediate actin assembly and disassembly through a RhoA-ROCK-dependent mechanism [89]. Filamin A regulates monocyte migration through Rho small GTPases during osteoclastogenesis [90]. FilGAP, a Rho-, and ROCK-regulated GAP for Rac binds filamin A to control actin remodelling [91]. Finally, endogenous FLNA is phosphorylated by PAK1 (an effector of Rac1 RhoGTPase) on ser2152 following stimulation with physiologic signaling molecules [92]. 

FLNA serves as a scaffold in intracellular cell signaling. For example, the actin-binding protein binds CMIP/TCMIP which are adaptor proteins involved in the T-cell signaling pathway [93]. It similarly regulates the activation of Sek-1, a dual-specificity protein kinase that is one of the immediate upstream activators of the stress-activated protein kinases (SAPKs) [78, 94]. FLNA interacts with Ship-2, a phosphatase that promotes insulin signaling [77]. 

Overall, FLNA integrates activation and signaling from various cell receptors, thereby mediating changes in actin dynamics through RhoGTPases and directing intracellular signaling of varied pathways. In this respect, the actin-binding proteins serve primarily as scaffolds that may bring together certain molecules within a given signaling pathway through actin assembly and disassembly. Classically, such a pathway involves extracellular matrix-dependent activation of integrin receptors, transduction of this signal through filamin onto PKC or FILGAP which directly promotes RhoA and RAC activation and subsequent changes in the actin cytoskeleton [95, 96]. 

### 5.4. Loss of FLNA Function in Mouse Model Studies

Prior studies have described vascular and skeletal abnormalities in null FlnA mice and in the Dilp2 mouse [97, 98]. The Dilp2 mouse harbors a T-to-A transversion that converts a tyrosine codon to a stop codon in the FlnA gene (Y2388X). Loss of FlnA function in either mice results in embryonic lethality at E15-16 with severe cardiac structural defects and midline fusion defects in the skeleton. 

FLNA is involved in neural progenitor proliferation. Loss of FLNA function in mice results in a thinned cerebral cortex. Moreover, loss of ventricular surface adherens junction proteins (i.e., VE-cadherin) along the basolateral surface suggests some disruption in neuroepithelial cell polarity and potentially, cytokinesis [98]. More recent findings demonstrate that the defect in proliferation is secondary to prolongation in the cell cycle, principally during M phase. FlnA loss impaired degradation of multiple cyclin-B1-related proteins, in part, through increased Cdk1 phosphorylation, thereby delaying the onset and progression through mitosis [99]. The delayed degradation in various cyclin-B-related proteins raises the possibility that FlnA might play a more prominent role in regulation of trafficking and stabilization of various molecules. Other studies have also shown that FLNA, like the Rho GTPase RhoA, is localized to the cleavage furrow [100], and that FLNA interacts with RhoA and both proteins are involved in regulation of actin. Taken collectively, these observations suggest that FLNA may play some role in cortical progenitor proliferation along the neuroependyma through RhoA- and actin-dependent trafficking of cell fate determining proteins.

FLNA is involved in initial neural progenitor migration. In early embryonic lethal (E13.5) null FLNA mice, migration into the cortical plate was ultimately preserved in neurons that had incorporated BrdU early in development [97, 98]. Other studies inhibiting FLNA function within neural progenitors along the ventricular lining by electroporation, however, have shown delayed migration into the cortical plate [101, 102]. Other studies have described the development of PH in the mitogen activated protein kinase kinase 4 (Mekk4) loss of function mice [49]. These mice also exhibit impairments in the onset of neural progenitor migration, disruption of the neuroependymal lining, and PH formation. Interestingly, FlnA levels were elevated in these mice, and Mekk4 appears to exist in a complex with Mkk4 and FlnA [78]. Overexpression of FlnA led to impairments in migration. More recent reports similarly suggest that the ADP-ribosylation factor guanine exchange factor 2 *Arfgef2* (encodes for the protein brefeldin inhibited guanine exchange factor 2, Big2) physically binds and interacts with FlnA. Loss of Big2 leads to upregulation of both FlnA and phospho-FlnA. Phosphorylation of FlnA alters FlnA binding affinity to actin and changes the size and distribution of paxillin focal adhesions, thereby altering cell autonomous migration into the cortex [103]. These findings suggest an interactive role between vesicle trafficking-related proteins and actin-associated proteins such as FlnA. 

## 6. *ARFGEF2* and PH

### 6.1. Genetic Phenotype

Mutations in *ARFGEF2* on chromosome 6 cause a rare autosomal recessive form of PH in humans. Affected individuals uniformly present with bilateral periventricular nodular heterotopia and microcephaly with generalized atrophy. Imaging studies tend to show hyperintensities in the basal ganglia. In one limited report, mutations in this gene are also associated with severe choreadystonic movement disorder [26, 29].

 The autosomal recessive PH due to mutations in the *ARFGEF2 *gene has been previously described in three pedigrees [26, 29]. The first pedigree displayed a missense mutation within a highly conserved amino acid (E209K). The second pedigree displayed a complex mutation (two missense mutations and one single nucleotide deletion) which predicted the premature termination of translation of the protein. The third pedigree showed compound heterozygosity for a duplication (base pairs 2031–2038) and deletion (base pairs 3798–3802) mutation, both of which led to a frameshift predicting a premature stop codon [29].

### 6.2. Genetic Structure and Function

Brefeldin A guanine exchange factor 2 (BIG2) is a 180 kDa protein which belongs to a family of three highly conserved large molecular weight mammalian Sec7-GEFs (GBF1, BIG1/2) [104]. They are distinguished by their sensitivity to the fungal metabolite Brefeldin-A (BFA) [105, 106]. The Sec7 domain is the most highly conserved region of the GEF family and is responsible for the GDP-to-GTP exchange and activation of ARFs [107–111]. BIG2 localizes along the Golgi and recycling endosomes and is thought to carry out ARF-dependent vesicle trafficking along these subcellular compartments [112]. Exo70, a member of the exocyst complex involved in vesicle exocytosis, also binds the N-terminal of BIG2 [37, 43]. Within its Exo70 binding region, several AKAP (A-kinase anchoring protein) binding sites are located. BIG2 residues 27–48 interact with PKA (protein kinase A) subunits RIalpha and RIbeta, residues 284–301 interact with subunits RIIalpha and RIIbeta, and residues 517–538 interact with subunits RIalpha, RIIalpha, and RIIbeta [113, 113]. Finally, at its C-terminal, BIG2 has been shown to bind the beta subunit of GABA receptors [114]. As a kinase anchoring protein, BIG2 is implicated in the spatiotemporal activation of cAMP signaling [115] and PKA-dependent phosphorylation also appears to regulate BIG2 activation of the ARFs [116]. Finally, as discussed below, Big2 has been shown to bind FlnA.

The ARFs are members of the Ras family GTPases involved in lipid vesicle budding from the membrane. They are generally myristoylated at the N-terminal domain to allow for membrane association and undergo cycling between the GTP and GDP-bound states. The active GTP form leads to a conformational change that exposes the myristate and hydrophobic N-terminal, thereby allowing for association with the membrane. The activated ARF binds to vesicle coat proteins and adaptors, including coat protein I (COPI) and various phospholipids. Guanine exchange factors (GEFs) such as BIG2 mediate ARF activation (GDP to GTP), whereas ARF GTPase activating proteins (GAPs) hydrolyze ARF-GTP back to ARF-GDP at the membrane. In the GDP-conformational state, the ARF becomes less hydrophobic and dissociates from the membrane. There are in total six ARF isoforms in mammals, which are grouped into three classes (Class I ARFs 1–3, Class II ARF4-5, Class III ARF 6) [117]. BIG2 has been shown to activate ARFs 1 and 3 *in vivo* through GTP activation[112]. ARF1/3 regulates cholera toxin-mediated endocytosis as well as endosome to endosome fusion. Moreover, dominantly active ARF1 can antagonize the inhibiting effects of Brefeldin A (an inhibitor of BIG2 function) [118, 119]. 

### 6.3. Loss of *Arfgef2* Function in Mouse Model Studies

The functional role of Big2 in the CNS is not well understood. BIG2 expression is developmentally regulated during cortical development and is most strongly localized to the neural progenitor cells along the neuroependymal lining of the ventricular zone during embryonic development. Expression of BIG2 is later downregulated during postnatal development and adulthood [120]. Recent work suggested early embryonic lethality in an *Arfgef2 *gene-trap mouse model, with insertion after exon 7. Fertilized eggs fail to develop after the four stage embryo [121]. This model, however, likely reflects a gain of Big2 function given that humans a second published transgenic model which creates a frameshift after exon 2, resulting in viable mice [103]. The loss of Big2 function mice develops heterotopia in the context of an underlying exencephaly which presumably disrupts the ventricular lining. These observations would suggest that PH is largely due to extrinsic disruption, likely of adhesion between cell-cell contacts along the neuroependyma. The mice also can exhibit midline closure defects, as seen with the FlnA mice. Finally, they do exhibit defects in neuronal migration, indicating some impairment in cell autonomous processes that regulate neuronal motility. However, this is not sufficient to cause PH.

## 7. General Mechanisms of Vesicular Trafficking

The endoplasmic reticulum (ER) is the primary site of lipid and protein synthesis in a cell, and vesicle budding from the ER provides a means with which to distribute proteins to their designated cellular location. These transport vesicles are defined by their lipid composition and protein coats, with coat proteins I/II (COPI/II) mediating protein/lipid export from the ER and Golgi. Rab GTPases are peripheral membrane proteins that are anchored into the membrane and regulate many steps of membrane traffic, including vesicle formation, vesicle movement along actin and tubulin networks, and membrane fusion. The composition of these various proteins mediates the transport to other endosome compartments or the plasma membrane [122, 123]. 

Endocytosis is a process characterized by four general mechanisms: clathrin-mediated endocytosis, caveolae, macropinocytosis, and phagocytosis [124]. Clathrin proteins form coated pits that cover the surface of an endosome. The coated pits concentrate large extracellular molecules that have different receptors responsible for the receptor-mediated endocytosis of ligands (i.e., transferrin and growth factors). On the other hand, caveolin-mediated endocytosis involves cholesterol binding caveolin proteins which form flask shape pits that resemble caves. Internalization is mediated by receptors within the caveolin laden lipid membranes. Pinocytosis involves the invagination of cellular membrane and subsequent uptake of smaller molecules and extracellular fluid into the endosome. Finally, phagocytosis involves cellular internalization of larger materials (greater than 0.75 microns including cellular debris, apoptotic cells, etc.) and is performed by specialized cells such as macrophages. 

Proteins synthesized in the ER traffic to the Golgi and then through specific endosomal compartment. When endocytosed, these compartment-specific proteins enter early endosomes and are sorted either to late endosomes, lysosomes, or degradation/recycling endosomes prior to return to the parent membrane. Alternatively, the proteins may undergo transcytosis; this process refers to the transfer of endocytosed proteins residing in one membrane (i.e., basolateral) that are sorted and modified in common endosomes and then transported to the opposite membrane (i.e., apical). The movement of vesicles is facilitated by the actin cytoskeletal network and associated motor proteins to maintain the apical and basolateral domains [125]. With delivery of the vesicle to the target lipid compartment, vesicle—SNARE (soluble N-ethylmaleimide sensitive factor attachment protein receptor) and target membrane—SNARE proteins bind to tether and assist in membrane fusion for cargo delivery. Additionally, the target lipid compartment (lipid rafts) often are composed of specific phospholipids that assist in signal transduction and as localized areas for vesicle fusion and delivery of specific protein cargo, such as the cell-cell junctional proteins (E-cadherin).

## 8. PH as a Defect in the Vesicle Trafficking Machinery

**Figure 1:**
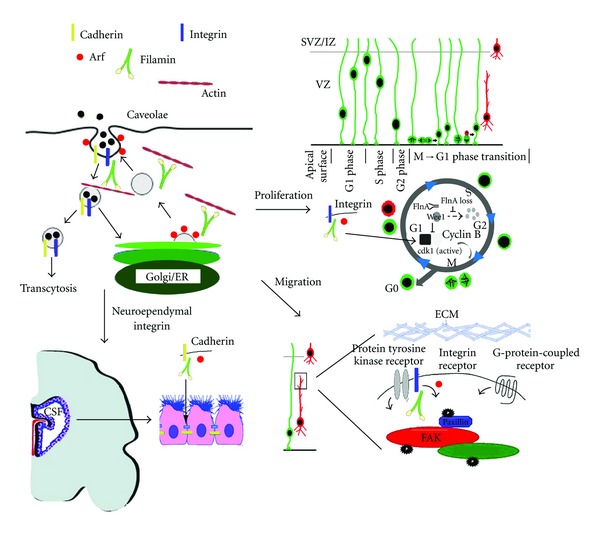
Periventricular heterotopia as a disorder of vesicle trafficking. Schematic figure illustrates the disruption of various developmental processes that give rise to features seen in PH. Disruption of filamin-ARF- (via BIG2) mediated vesicle trafficking can disrupt both caveolin-mediated endocytosis and presumably trafficking from the Golgi to the membrane. Vesicle trafficking dictates the delivery and degradation of various cell cycle proteins and thereby influences cell proliferation. Disruption of the cyclin B proteins leads to microcephaly in PH. Vesicle trafficking also alters the distribution of proteins involved in neuronal migration such as paxillin. Loss of PH impairs neuronal migration and the morphology of migratory neurons. Lastly, the loss of neuroependymal integrity giving rise to heterotopia is due to impairments in the cell adhesion along the ventricular lining. Genes that disrupt vesicle trafficking impair cell-cell adhesion and disrupt the integrity of the epithelium.

An overriding defect in the vesicle trafficking machinery contributes to PH formation (Figure 1). The characteristic heterotopic nodules found along the ventricular lining are thought to arise from a disruption in cell-cell adhesion along the neuroependymal lining as well as a defect in initial neuronal migration [25, 101, 103]. Both FLNA and BIG2 expression are highly regulated along the neuroependymal surface and within neural progenitor cells during embryonic development [120], and both proteins are implicated in vesicle trafficking. FLNA dynamically associates with Golgi and vesicle membranes and regulates the trafficking of membrane proteins (furin, transferrin receptors) as well as caveolin [126, 127]. BIG2 regulates ARF-dependent vesicle budding along the Golgi and endosomal membrane compartments [112]. Recent work suggests that Big2 binds FlnA and regulates its level of phosphorylation [103]. The inhibition of FLNA or BIG2 function also leads to similar defects in the trafficking of adherens junction proteins (*β*-catenin, E-cadherin). Overexpression of dominant negative mutant FLNA (actin-binding deficient) within neural progenitor cells along the ventricular surface leads to the mislocalization of *β*-catenin from the basolateral cell membrane [25]. In addition, mice with null mutations in *Flna *gene display the loss of vascular endothelial cadherin along the neuroependymal surface [98]. Similarly, overexpression of dominant negative mutant BIG2 (Sec7-inactivated) leads to the abnormal membrane localization of *β*-catenin and E-cadherin in polarized Madin Darby Canine Kidney cells (MDCK) [26]. Intraventricular injection of brefeldin-A leads to the loss of adherens junction proteins (*β*-catenin, N-cadherin) along the neuroependymal surface and the denudation of the ventricular surface followed by PH formation [25]. Overall, these findings suggest that an interaction between actin (FlnA) and vesicle budding (Big2) lead to destabilization of the lipid membrane, impairments in cell adhesion molecules, and loss of ependymal integrity.

Mouse genes causal for PH collectively suggest a defect in vesicle trafficking in this disorder and appear to be involved in filamin-dependent pathways. Alpha SNAP is a SNARE-related protein, involved in vesicle fusion. Prior reports have shown that alpha SNAP mediates VE-cadherin localization through a b1-integrin-associated process [128]. FlnA binds b1-integrin. Mekk4 is a regulator of FlnA and therefore indirectly regulates caveolin or clathrin-mediated endocytosis. The rhoGTPases bind FlnA and direct various aspects of intracellular actin dynamics, which are required for endosomal vesicle transport. In a similar manner, Spred1 is a multidomain scaffolding protein that contains an ENA/VASP domain that can modulate actin stress fiber remodeling, in a manner similar to the filamins. Spred1 is also associated with specific endosomal vesicles [48]. Finally, SCF-c-kit effects several downstream pathways including RAS/ERK and JAK/STAT pathways, both of which have been associated with Mekk4 and Spred1 activity [129].

Although disruption in the neuroependymal lining may be the primary cause of heterotopia formation, altered vesicle trafficking would be expected to contribute to other CNS and extra-CNSanatomicaldefects. Several studies have demonstrated impaired neuronal migration and motility following inhibition or overexpression of FlnA or Big2 function [49, 101–103]. For example, loss of Big2 function leads to upregulation of FlnA phosphorylation and changes in paxillin cluster size and number in migratory neurons [103]. As vesicle trafficking reflects a homeostatic process, both increases and decreases in trafficking-associated proteins could lead to changes in thelocalization and stability of molecules associated with neuronal migration. Loss of FlnA function alsoleads to a reduction in brain sizesecondary to prolongation in M to G2 phase progression of neural progenitorsand delayed clearance of various cyclin-B-associatedproteins [99]. The impaired degradation of theseproteins again suggests somedefect in vesicle trafficking. Not unexpectedly, these defects in progenitor proliferation also appear to affect chondroprogenitors and intestinal stem cells,asloss offilamin function leads toskeletal midline closure defects and a shortened gut. Loss of integrity of the liningcanalsobe appreciated in blood vessels where the embryonic lethality of null FlnA mice isdue to a vasculopathy and bleeding frombreakdown of the endothelial lining [58, 98].

## 9. Future Directions

The ventricular neuroepithelium of the cerebral cortex is a dynamic structure, whose maintenance depends on the continuous turnover of polarized vesicle trafficking. During cortical development, neuroepithelial cells (neural progenitors) give rise to all the neurons and astrocytes of the cerebral cortex. The cell fate decision of progenitors from self-renewal to differentiation is a crucial factor in determining the morphology and size of the brain. Cell fate specification is regulated by symmetric and asymmetric divisions, which are further governed by mitotic spindle orientation. Vesicle trafficking maintains the apical-basal polarity of neuroepithelium through directional vesicle transport and vesicle sorting, thereby dictating cell polarity and symmetric/asymmetric division. These mechanisms will also dictate the integrity of the neuroepithelium, the direction and rate of neuronal migration, as well as connectivity between neurons. While FLNA and BIG2, as well as various mouse genes, have been implicated in endosomal vesicle trafficking and defects in this pathway can give rise to the various neural defects, the downstream effectors of these processes are not know. It will be important to determine which actin effectors (besides the RhoGTPases) are responsible for changes in actin dynamics through FLNA and which endocytic vesicles (caveolin, clathrin, etc.) and the stage of endocytosis (early, late, lysosomal, etc.) through BIG2 are responsible for the various phenotypes. Lastly, the direct interplay between actin and vesicle trafficking is poorly understood and studies going forward will be aimed at addressing this biological process.

## 10. Conclusions

Vesicle trafficking plays a central role through the various stages of cortical development, and disruption of these mechanisms contributes to different aspects of cortical malformations. Cyclin-associated proteins regulate the rate of progression through the cell cycle and thereby dictate neural progenitor proliferation. The turnover of cyclin-associated proteins is dependent on vesicles and impaired degradation leads to prolongation of the cell cycle and a reduction in brain size. During neural progenitor expansion cells translocate from the neuroependymal lining toward the cortical surface and back again. Neuroependymal integrity is maintained by cell-cell junctions, which are dependent on vesicle transport. Disruption of the neuroependymal lining causes displacement of progenitors near the ventricular lining and PH formation. Next, migrating neurons must travel from the ventricular zone to the cortical plate. The leading and trailing processes of migrating neurons require active turnover of lipid membranes and focal adhesion-associated proteins for proper motility; disruption of these vesicle-dependent processes results in impairments in neuronal migration. Although unproven, it seems likely that the dyslexia and seizures associated with genes cause for PH will also reflect vesicle trafficking-related problems in synaptic connectivity. Thus, while disorders of vesicle trafficking may be linked to PH formation also, these fundamental processes give rise to a much broader cortical phenotype than previously realized.
